# Unusual Gingival Enlargement: A Rare Case Report

**DOI:** 10.1155/2014/536312

**Published:** 2014-03-16

**Authors:** Ashutosh Dixit, Seema Dixit, Pravin Kumar

**Affiliations:** ^1^Department of Periodontics, Seema Dental College, Rishikesh, India; ^2^Department of Conservative Dentistry and Endodontics, Seema Dental College, Rishikesh, India

## Abstract

This is an atypical case report of a 20-year-old male patient who suffered from unusual unilateral, gingival enlargement together with rapidly progressive alveolar bone loss. The enlarged gingiva completely covered his left posterior teeth in both arches. The patient was diagnosed with gingival fibromatosis and aggressive periodontitis based on the clinical, histological, and radiographic findings. The gingival enlargement was treated by conventional gingivectomy under local anaesthesia. The postoperative result was uneventful.

## 1. Introduction

Gingival fibromatosis, gingivomatosis [1], diffuse fibroma [2], familial elephantiasis [3], idiopathic fibromatosis, hereditary gingival hyperplasia, gigantism of gingiva, and hypertrophic gingiva are slowly progressive fibrous enlargements of the maxillary and mandibular gingiva.

Gingival fibromatosis may be an inherited condition as in hereditary gingival fibromatosis or may be associated with medications or may be idiopathic. It may also be caused by inflammation or leukemic infiltration.

The gingival fibromatosis may occur as an isolated finding or be associated with one of several hereditary syndromes, for example, Rutherford's Syndrome, Jones' Syndrome, Murray-Puretic-Drescher Syndrome, Laband Syndrome, Ramon's Syndrome, hypothyroidism, and so forth [4]. The syndromic characteristic most commonly seen in association with hereditary gingival fibromatosis is hypertrichosis [5]. It is characterized by massive gingival enlargement that appears to cover the tooth surfaces. The enlargement may be associated with one or more teeth, involve one or more quadrants, or may be generalized. The cause of enlargement is unknown but there appears to be a genetic predisposition.

Gingival hyperplasia produces conditions favorable for the accumulation of plaque and materia alba by accentuating the depth of gingival sulcus and by interfering with effective hygiene measures. The secondary inflammatory changes further increase the size of the preexisting gingival hyperplasia [6].

Aggressive periodontitis is a genetically inherited disease that represents a severe and rapidly progressive form of periodontitis [7]. This form of periodontitis presents peculiar clinical presentation, occurring around puberty with an apparent lack of local factors such as heavy amounts of plaque and calculus in patients with reasonably good oral hygiene.

Aggressive periodontitis is characterized by the following major common features:

1. early age of clinical manifestation,
2. noncontributory medical history,
3. rapid attachment loss and bone destruction,
4. familial aggregation.

This paper presents a rare case of a nonsyndromic idiopathic gingival enlargement associated with aggressive periodontitis and its management.

## 2. Case Report

A 20-year-old male patient reported at the Department of Periodontology, Seema Dental College and Hospital, Rishikesh, with chief complaint of gingival swelling around left upper and left lower posterior teeth and inability to chew food from the left side. Patient gave history of trauma to the left side of his face three months ago after which the gingival swelling first appeared and gradually increased to its present size. However, no correlation was found between trauma and gingival enlargement. The patient came for treatment only when the swelling started interfering with mastication. Medical history was non contributory in this case. He could not provide adequate family history as he was illiterate and was not able to provide the details.

**Figure 1:**
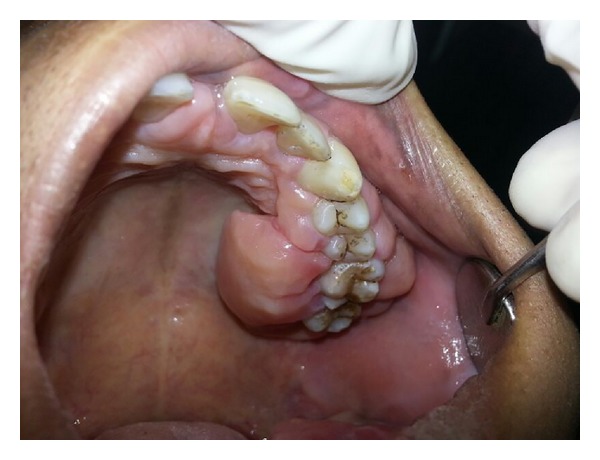
Gingival hyperplasia on left side of maxillary arch.

**Figure 2:**
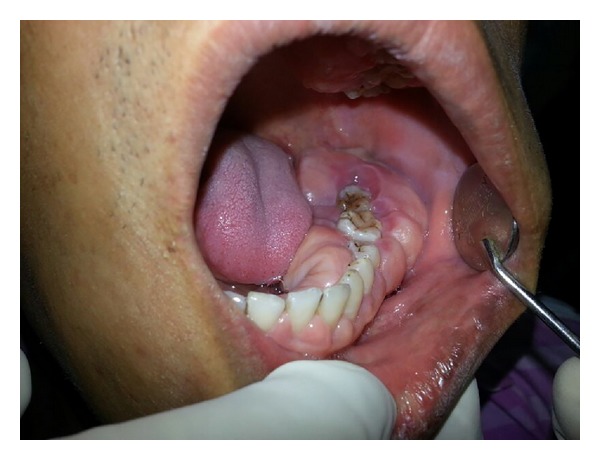
Gingival hyperplasia on left side of mandibular arch.

Upon intraoral examination, gingiva seemed to be grossly enlarged on left side. The gingival enlargement extended from first premolar to second molar region in both maxillary arch (Figure 1) and mandibular arch (Figure 2). The enlargement was firm and fibrotic accompanied by an inflammatory component probably due to his inability to maintain adequate personal oral hygiene. The gingival enlargement extended from the buccal to the lingual palatal mucosa. The diffuse type of enlargement involved marginal, interdental, and attached gingiva. The periodontal probing revealed deep pockets in relation to the involved teeth. Mobility was also present in all the involved teeth.

### 2.1. Radiological Examination

**Figure 3:**
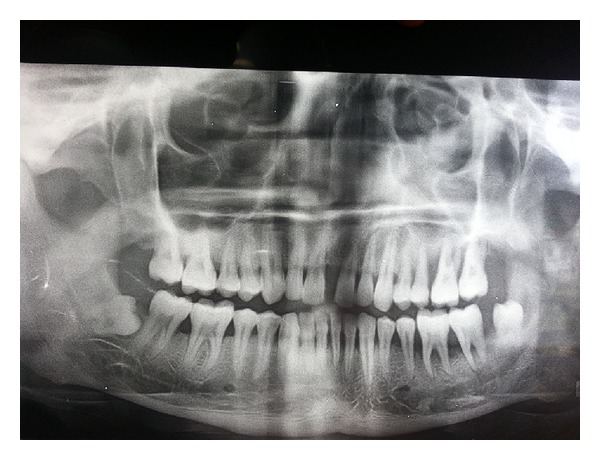
OPG.

Panoramic view revealed presence of remaining bone in the range of 30 to 35% on left side of both arches. Bone loss was severe in relation to maxillary left first molar and mandibular left first molar (Figure 3).

### 2.2. Hematological Examination

Routine hematological investigations revealed hemoglobin 12.6% and differential leucocyte count of polymorphonuclear leukocytes 75%, lymphocytes 23%, monocytes 0%, eosinophils 2%, and basophils 0%.

### 2.3. Histological Examination

Incisional biopsy was performed which showed stratified squamous epithelium overlying the connective tissue stroma. In few areas, flattening of the rete ridges was seen whereas in other areas they appeared elongated. The connective tissue appeared densely collagenized and was seen to be interspersed with plump- and spindle-shaped fibroblasts. Few chronic inflammatory cells mainly consisting of lymphocytes were seen dispersed throughout the section. Areas of small- to medium-sized endothelial lined blood vessels were also evident (Figure 9).

Based on the above findings, the diagnosis of gingival fibromatosis along with aggressive periodontitis was established.

### 2.4. Treatment

**Figure 4:**
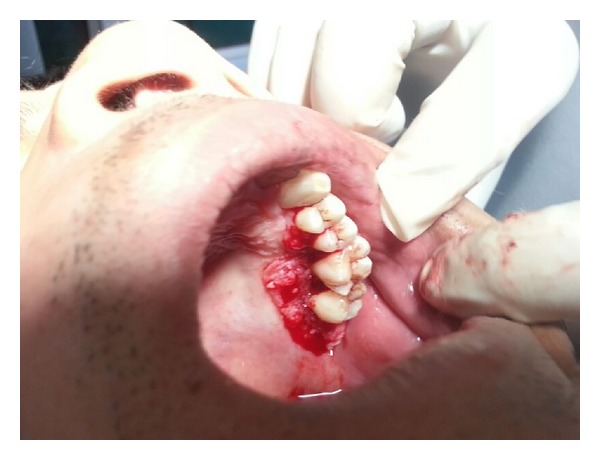
Operative (maxillary).

**Figure 5:**
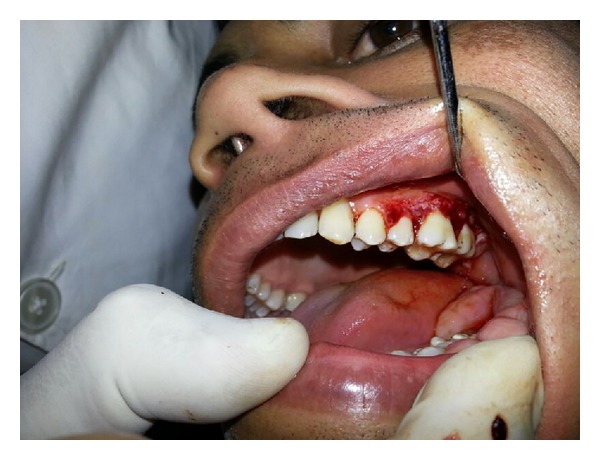
Operative (maxillary).

**Figure 6:**
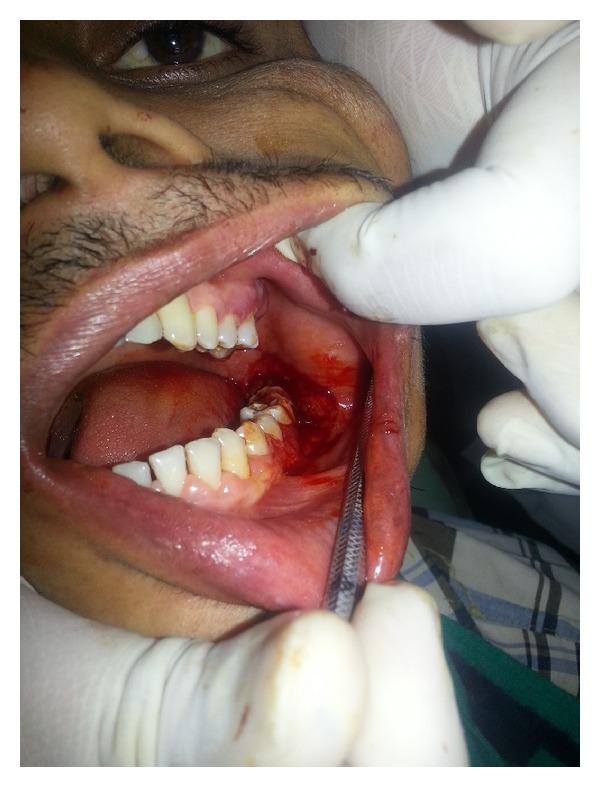
Operative (mandibular).

Initial therapy comprised of conventional periodontal therapy. The enlarged tissue was removed by the external bevel gingivectomy under local anesthesia. The surgery was performed in two stages, left maxillary arch in the first stage (Figures 4 and 5) and left mandibular arch in second stage (Figure 6). The gap between the two procedures was three weeks. Periodontal dressing was applied. Antibiotics, fortified B-complex, and anti-inflammatory agents were prescribed for one week.

**Figure 7:**
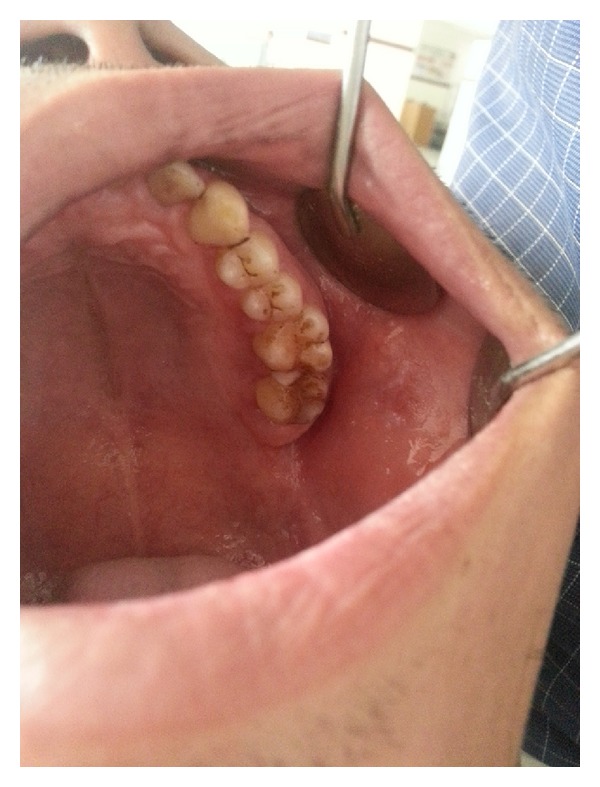
One month post operative view (maxillary).

**Figure 8:**
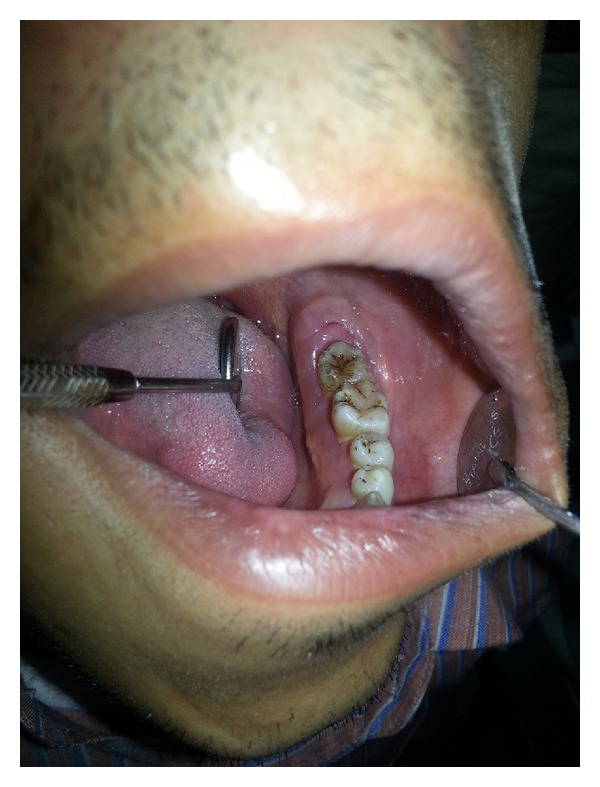
One month post operative view (mandibular).

**Figure 9:**
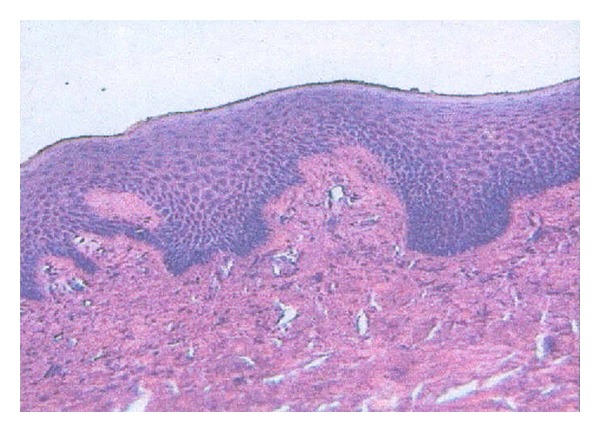
Microscopic appearance.

Postsurgical healing was uneventful. Periodontal dressing was removed after one week. The surgical area was generously irrigated with betadine and normal saline. Postoperative instructions were reinforced and patient was recalled after one week and then after one month for postsurgical evaluation. Healing was uneventful after one month (Figures 7 and 8). Patient was satisfied with the result.

### 2.5. Discussion

Gingival fibromatosis is seen frequently to be associated with various syndromes. It has been associated with Rutherford's Syndrome, Laband Syndrome, Cross Syndrome, Murray-Puretic-Drescher Syndrome, Jones' Syndrome, hypertrichosis, and epilepsy. It can also be caused by a number of factors, including inflammation, leukemic infiltration, and use of medications such as phenytoin, cyclosporine, or nifedipine.

This paper reports a case of severe gingival enlargement on left posterior region in both arches. Blood investigation did not reveal any abnormality. The clinical and histological features and systemic examination excluded the diagnosis of neoplastic enlargement. Gingival hyperplasia occurs in some patients taking certain drugs such as phenytoin, cyclosporine, and nifedipine. The patient had not taken any of these drugs.

The patient was normal without any symptoms of mental retardation. He did not suffer from epilepsy or hypertrichosis nor did he have any tumors or corneal dystrophy. No skeletal deformities or defects of the skin or fingernails were observed. Thus, it is unlikely that the syndromes mentioned earlier were related to this gingival enlargement.

In many types of gingival enlargement, the rapid loss of alveolar bone or attachment level is not observed. However, this patient exhibited rapidly progressive destruction of the periodontal tissues. Alveolar bone loss was observed in his oral radiograph. There was a lack of clinical inflammation despite the presence of deep periodontal pockets and advanced bone loss. The amount of plaque on the affected teeth was minimal, which seemed inconsistent with the amount of periodontal destruction present.

In this case, the patient is a young 20-year-old male, with a noncontributory medical history and severe bone destruction relating it to the diagnosis of aggressive periodontitis. Regarding familial aggregation, patient was not able to explain properly about family members as he belongs to a nearby village and was an illiterate person.

The first case of this kind was reported in 2004 by Casavecchia et al. [8]. Before 2004, no such case was reported. Historically, gingival fibromatosis has been reported to be an associated finding with various other syndromes or disorders but has not been reported in coexistence with aggressive periodontitis. They performed genetic analysis as well as analysis of neutrophil function but could not establish the common mechanism, if any, that could be involved in the pathogenesis of these two entities.

After Casavecchia et al., three such cases have been reported where there was nonsyndromic, idiopathic gingival enlargement associated with generalized aggressive periodontitis [9–11].

Aggressive periodontitis is typically characterized by familial aggregation because of evidence of genetic predisposition that was derived from segregation analysis of affected families. Mendelian inheritance occurs and autosomal (dominant and recessive) transmission and X-linked transmission have been proposed [11]. This patient had a diagnosis of generalized aggressive periodontitis with idiopathic gingival fibromatosis based on his clinical findings and no significant family history.

The results of the histopathologic evaluation in this case revealed the features of fibrous gingival hyperplasia, that is, the presence of a thickened acanthotic epithelium with elongated rete ridges and densely arranged collagen bundles with numerous fibroblasts and with some areas of neovascularization.

The treatment need varies according to the degree of severity; when the enlargement is minimum, thorough scaling of the teeth and home care may be essential to maintain good oral health. The relative increase in the gingival mass contemplates the need for surgical intervention. In the past, treatment included extraction of all involved teeth and reduction of alveolar bone. Many techniques have been used for excision of enlarged gingival tissues including external or internal bevel gingivectomy in association with gingivoplasty, an apically positioned flap, electrocautery, use of twelve fluted carbide bur in high speed hand piece, and CO_2_ laser [5]. However, the most widely used method of removing large quantities of tissue is the conventional external bevel gingivectomy [12]. Recurrence is a common feature over varying periods. In several reported cases, there was no recurrence in a period of 2 years [13], 3 years [14], or even 14 years [15] of followup.

For the present case, external bevel gingivectomy under local anaesthesia was done in both maxillary and mandibular arches. Healing was uneventful and patient was satisfied. Patient was told about recurrence and was instructed to follow routine oral hygiene measures strictly to prevent recurrence.

## 3. Conclusion

This is a rare case presentation of a true combined lesion of gingival enlargement with generalized aggressive bone destruction, which is not a common feature seen together. Diagnosis was based primarily on clinical radiographic and histopathologic assessments. However, further research including immunological and genetic testing is needed to establish association between the two conditions.
